# Young pharmacists as tomorrow’s decision-makers: tool validation and perceptions of pharmaceutical policymaking in Lebanon

**DOI:** 10.1080/20523211.2025.2600242

**Published:** 2025-12-23

**Authors:** Chadia Haddad, Hala Sacre, Jihan Safwan, Deema Rahme, Aline Hajj, Jenny Elia, Joya El Ghawi, Lina Haidar, Lama Dimachkieh, Mahmoud Nasrallah, Soukaina Basma, Pascale Salameh

**Affiliations:** a Institut National de Santé Publique, d'Épidémiologie Clinique et de Toxicologie-Liban (INSPECT-LB), Beirut, Lebanon; b Research Department, Psychiatric Hospital of the Cross, Jal Eddib, Lebanon; c Faculty of Public Health, Lebanese University, Fanar, Lebanon; d School of Pharmacy, Lebanese International University, Beirut, Lebanon; e Pharmacy Practice Department, Faculty of Pharmacy, Beirut Arab University, Beirut, Lebanon; f Faculté de Pharmacie, Université Laval, Québec, Canada; g Oncology Division, CHU de Québec – Université Laval Research Center, Quebec, Canada; h Lebanese Pharmacy Students’ Association, Beirut, Lebanon; i Faculty of Pharmacy, Lebanese University, Hadat, Lebanon; j Gilbert and Rose-Marie Chagoury School of Medicine, Lebanese American University, Beirut, Lebanon; k Department of Primary Care and Population Health, University of Nicosia Medical School, Nicosia, 2417, Cyprus

**Keywords:** Policymaking, strategic thinking, leadership, public service motivation, general self-efficacy, pharmacy

## Abstract

**Background:**

The perception of pharmacy policymaking among early-career pharmacists is crucial for developing and advancing the profession. This study aimed to construct and validate a new tool, the Pharmaceutical Policymaking Perception Scale (PPPS), and assess pharmacy students’ and graduates’ perceptions of pharmaceutical policymaking in Lebanon.

**Methods:**

A standardized questionnaire was disseminated through electronic platforms. It included sociodemographic characteristics, education-related variables, and scales measuring leadership, general self-efficacy, strategic thinking, and public service motivation. The validity of the newly developed PPPS was confirmed, and concepts were linked through multivariate analyses.

**Results:**

The PPPS tool exhibited excellent psychometric properties, with its items loading on two factors representing the positive and negative perceptions of pharmaceutical policymaking. The scale demonstrated excellent reliability as well as robust content, construct, structural, and concurrent validity. Only 4% of participants scored above 70, indicating relatively low perceptions of pharmaceutical policymaking in Lebanon. Higher PPPS scores were associated with higher self-efficacy and strategic thinking, while lower scores were linked to reduced public service motivation. No association was found between PPPS and leadership.

**Conclusion:**

The novel PPPS scale offers valuable insights into pharmacists’ views, enabling a more comprehensive assessment of policymaking perceptions. The potential disconnection between the studied concepts raises concerns. Further research is recommended to confirm these findings, and urgent action by educators and policymakers is essential to effectively engage with early-career pharmacists and enhance their motivation to serve the profession in challenging circumstances.

## Background

Pharmacists play a crucial role in healthcare and must contribute to its advancement at the global, regional, and national levels. Their responsibilities should extend beyond community pharmacies to include participation in pharmaceutical policymaking. This involvement is essential to improving access to medication, ensuring optimal care, and advocating for patients’ rights (Morrow, 2015; Mossialos et al., 2013; Patwardhan et al., 2012; Traulsen & Almarsdóttir, 2005).

Pharmacists, as drug experts, have valuable knowledge and experience that should not be overlooked in policymaking. However, research indicates that most pharmacists are not involved in pharmaceutical policymaking (Morrow, 2015). Their knowledge of drug therapy, interactions, and medication management makes them well-positioned to contribute to the development of informed laws and policies (Morrow, 2015). Furthermore, incorporating pharmacists’ knowledge and experience into policymaking is fundamental to the continuity of the pharmacy profession and the healthcare industry in general (Morrow, 2015; Traulsen & Almarsdóttir, 2005). This inclusion will lead to the development of laws and regulations better aligned with the needs of patients and the healthcare system, ultimately improving patient care and advancing the profession (Morrow, 2015; Traulsen & Almarsdóttir, 2005).

Moreover, the development and sustainability of the pharmacy profession in Lebanon require an evaluation of the policy understanding, future policymaking, and leadership abilities of Lebanese pharmacy students. Challenges, such as the need for policymaking, financial worries, and pharmacist dissatisfaction (Alameddine et al., 2019; Hallit et al., 2017; Iskandar et al., 2017; Salameh & Hamdan, 2007), highlight the need to educate proficient policymakers and leaders within the profession. Although pharmacy students have limited involvement in positions requiring leadership skills, these competencies are vital for handling the challenges faced by pharmacy professionals (Ali et al., 2022), particularly in negotiating legislation changes, advocating for the industry, and safeguarding the health of practitioners and patients. Furthermore, strategic thinking is indispensable for foreseeing and effectively managing the changing healthcare landscape (Persky et al., 2019).

The pharmacy profession in Lebanon faces now a multifaceted crisis requiring urgent attention and innovative solutions. With a pharmacist-to-population ratio of 20.3 per 10,000, one of the highest globally, the country is grappling with a severe oversupply of graduates (Alameddine et al., 2019, p. 2020). This significant demand-supply imbalance has led to the closure of hundreds of pharmacies and widespread unemployment among pharmacists (Hallit et al., 2019). Adding to these issues are outdated regulatory policies that are poorly enforced, fostering unethical practices and eroding public trust in the profession (Alameddine et al., 2019, p. 2020). The economic meltdown in Lebanon has further exacerbated these challenges, causing drug shortages and financial instability for pharmacies (Alameddine et al., 2020). In this critical landscape, there is an urgent need for innovative policymaking, led by pharmacists with robust leadership skills, strategic thinking capabilities, and a deep sense of public service motivation. These professionals must be equipped to develop and implement novel strategies to revitalize the pharmacy sector, optimize workforce distribution, and enhance the profession’s role in public health (Hajj et al., 2022). In recent years, the scientific committee at the Order of Pharmacists of Lebanon (OPL), in collaboration with academic researchers and other pharmaceutical stakeholders, developed a National Pharmaceutical Strategy (Sacre et al., 2023), followed by educational, workforce, and research strategies (Akel et al., 2022; Hajj et al., 2023). However, the ongoing challenges in the country hinder the implementation of these initiatives. By addressing these obstacles, pharmacy authorities could steer the pharmacy profession in Lebanon toward a more sustainable and impactful future.

Pharmacists must possess several characteristics to lead in policymaking (Persky et al., 2019). These include critical thinking skills (such as questioning, interpreting, analyzing, and synthesizing information, as well as self-regulation and metacognition), strategic thinking abilities (creating a vision, developing and implementing strategies, and making informed decisions), leadership competencies (communication skills, especially for inclusive and person-centered care, cultural competence and safety, relationship-building with diverse health consumers, and project management capabilities), professional skills (problem-solving, clinical decision-making, self-directed learning, initiative, creativity, innovation, reflection, and self-awareness), and specialized knowledge of the healthcare system.

The Competency Framework for Pharmacy Education and Practice served as the conceptual framework for this study (Hajj et al., 2021). It includes seven core domains (fundamental knowledge, professional practice, personal skills, medicines supply, safe and rational use of medicines, pharmaceutical public health, and organization and management), comprising 35 competencies and 297 behaviors. It offers a structured approach to articulating the fundamental skills expected of pharmacy graduates for effective practice, including leadership and policymaking. This framework has been previously adopted by the Order of Pharmacists of Lebanon (OPL) to guide educational reforms and align local pharmacy competencies with international standards. It also aligns directly with the constructs explored in this study: leadership, self-efficacy, strategic thinking, and public service motivation.

### Objectives

This study aimed to construct and validate a new tool, the Pharmaceutical Policymaking Perception Scale (PPPS), among Lebanese pharmacy students and graduates. It also sought to assess participants’ perceptions of pharmaceutical policymaking in Lebanon, focusing on leadership, general self-efficacy, strategic thinking, and public service motivation after validating the related scales among a sample that included both pharmacy students and professionals. The inclusion of both groups was intended to capture general trends across the pharmacy field in Lebanon, regardless of career stage.

By examining these concepts, the study could provide valuable insights into the willingness of the new generation of pharmacists to serve the profession while appraising their skills and potential interests. The topics explored in this research are pivotal to the development and advancement of the pharmacy profession.

## Methods

### Study design and sampling

This cross-sectional study was conducted from December 2023 to May 2024 among pharmacy students in Lebanon. The online questionnaire was created on Google Forms and distributed conveniently to all participants. Students were reached through the Lebanese Pharmacy Students’ Association (LPSA), while early-career pharmacists (those who graduated less than ten years ago) were targeted via various social media platforms. The LPSA having exhaustive lists and social media groups that include the pharmacy programs’ university students and recent graduates from Lebanon, and the survey was sent through an electronic link to all members. The procedure was repeated more than 10 times to reach the necessary sample size for the current study. In parallel, the authors contacted instructors from all universities and asked them to distribute the survey to their students. Additional social media platforms were used to further diffuse the survey to pharmacy graduates (Facebook, Instagram and LinkedIn).

All essential questions in the Google Forms survey were set as mandatory, with response validation rules implemented to minimize entry errors and maintain data quality. All items were carefully worded to maximize clarity and reduce the risk of misinterpretation. The form was designed to collect responses anonymously, with no email addresses or other identifiable information recorded. Access to the form and the associated Google account was restricted to the principal investigator, and data were downloaded and stored securely offline. Given the questionnaire’s length, it was deemed unlikely that the same participant would complete it twice, eliminating the need for additional duplicate detection measures.

### Data collection tool

The standardized questionnaire was available in English and covered sociodemographic characteristics, education-related variables, and questions about leadership, general self-efficacy, strategic thinking, and policymaking. The sociodemographic characteristics could be linked to personal characteristics, such as strategic thinking and leadership (Wiersema & Bantel, 1992), while the educational characteristics are expected to correlate with all the measured concepts through competencies frameworks that ground the educational programs adopted by various universities in Lebanon (Hajj et al., 2021).

### Development of the Pharmaceutical Policymaking Perception Scale (PPPS)

As for the pharmaceutical policymaking perception, questions related to pharmacy students’ and graduates’ opinions about current leadership in pharmacy were asked. The content of this tool was validated by an expert committee that included six experts (authors of the manuscript). They joined efforts to evaluate the content of the suggested questionnaire; five are previous members of the OPL scientific committee, four are also academic pharmacists (two mid-career and two seniors), and one is a managing director at the OPL. All have been involved in research on various aspects of pharmacy policies, education, workforce, and practice. In addition, four pharmacy students and early graduates from the Lebanese Pharmacy Students Association (LPSA) joined the research team and gave their input. Face validity was established through expert agreement that the items appeared to adequately reflect the domains of interest. Content validity was ensured through iterative item refinement based on expert feedback, with adjustments made to wording and scope to enhance alignment with the study objectives and the context of pharmaceutical policymaking in the country.

The PPPS scale content was thus validated using a Delphi technique, where a consensus of 90% was reached for every included question; a total of 13 questions were included in the final form of the scale. The questionnaire includes a series of items developed through a comprehensive literature review that directly assess participants’ perceptions toward pharmaceutical policies and policymakers in the country. These items explore dimensions such as the timeliness, evidence basis, leadership, and effectiveness of policy decisions, as well as perceptions of policy implementation and the role of pharmacists in policy processes. The questionnaire might be a useful tool for identifying perceptions toward pharmaceutical policy and policymaking areas for improvement.

Afterward, the developed tool was included in the overall questionnaire for further validation: reliability, construct validity, structural validity, concurrent validity, and known group validity. One additional question was further removed (Question 4: Policymakers make non-informed decisions) from the scale due to low communality (<0.3).

### Cross-cultural validation of the used tools

The following scales were used to assess leadership, general self-efficacy, strategic thinking, and public service motivation. Due permissions were obtained from the scale developers before use. As all pharmacists and pharmacy students understand English in Lebanon, the scales were not translated into Arabic. As recommended by the expert panel, they were kept as such, and their structure was validated in the current sample (results reported in Supplemental Material).

#### The authentic leadership self-assessment questionnaire (ALSAQ-16)

This 16-item tool is designed to assess authentic leadership using a 5-point Likert scale from strongly agree to strongly disagree (Gardner et al., 2013; Panczyk et al., 2019). It has three unidimensional subscales (moral processing, self-awareness, and relational transparency), and a high reliability (Cronbach's alpha = 0.84).

#### The general self-efficacy scale (GSE-10)

The GSE is a 10-item self-reported scale designed to assess optimistic self-beliefs to cope with the various demands of life by referring to oneself, i.e. the belief that a person’s actions are responsible for successful outcomes (Schwarzer & Jerusalem, 1995). Items are rated on a 4-point Likert scale, with higher scores indicating higher self-efficacy.

#### The strategic thinking questionnaire (STQ-15)

This 15-item tool assesses strategic thinking skills by asking the respondents to rate how often they used these skills when faced with problems, dilemmas, or opportunities on a Likert-type scale, with higher scores representing greater use of cognitive skills (Pisapia et al., 2011).

#### The public service motivation scale – short version (PSM-14)

The short version of Perry’s PSM was used in this study (Perry, 1996). It includes 14 items measuring three dimensions, i.e. self-sacrifice, attraction to policymaking, and compassion, on a 5-point Likert scale from strongly disagree to strongly agree (Gan et al., 2013). Higher scores indicate higher public service motivation.

### Minimum sample size calculation

The minimum sample size was calculated using the G-Power software, version 3.0.10. The calculated effect size was 0.0526, expecting a squared multiple correlation of 0.05 (R^2^ deviation from 0) related to the omnibus test of multiple regression. The minimum necessary sample was n = 454, considering an alpha error of 5%, a power of 80%, and allowing 25 predictors to be included in the model. A minimum sample of 500 participants was targeted to account for potential missing values and enable the validation operation (a minimum of 10 participants needed per item) (Nunnally & Bernstein, 1994).

### Statistical analysis

The collected data were analyzed using SPSS (Statistical Package for Social Sciences) version 28.0. Since this is the first time these scales are used among Lebanese early career pharmacists, the scales were first validated using exploratory factor analysis, with additional assessments of structural, concurrent, and divergent validity using Spearman coefficients. Reliability (internal consistency) was evaluated using Cronbach’s alpha. These analyses support the internal consistency and dimensionality of the instrument within the Lebanese context.

Descriptive analysis included frequencies and percentages for categorical variables, while means and standard deviations were calculated for quantitative variables. Medians and interquartile ranges were also reported. The distribution of quantitative variables was evaluated for normality by visual inspection of histograms, with skewness and kurtosis values below 1. These conditions and a sample size exceeding 300 were considered compatible with normality across all scales.

Bivariate analysis of continuous variables was conducted using the Student’s t-test to compare means between two groups and ANOVA for comparison between three or more groups after assessing homogeneity of variances via Levene’s test. When variances were not homogenous, the corrected t-test and Kruskal–Wallis test were applied, respectively. Post hoc analyses were performed using Bonferroni adjustment after significant ANOVA and Kruskal–Wallis results. Spearman correlation coefficients were calculated for relationships between scale variables. For categorical variables, the Chi-squared test was used, and the Fisher exact test was applied when expected cell counts were less than 5. In all cases, a *p*-value of less than 0.05 was considered statistically significant.

For the multivariable analysis, multiple linear regressions were conducted using the UNIVARIATE ANCOVA GLM procedure to assess the correlates of dependent variables across the entire sample. Assumptions of normality of residuals, linearity of relationships, absence of multicollinearity, and homoscedasticity were verified before analysis. The ENTER method was used to include all potential confounding variables in the models. Additionally, a multivariate MANCOVA was conducted to account for multiple dependent variables. Independent variables introduced in the models were those identified in the bivariate analysis, along with relevant sociodemographic and other independent variables as appropriate. Results were reported as beta coefficients with their 95% confidence intervals and corresponding *p*-values.

## Results

Table 1 presents the descriptive characteristics of the participants. The majority were female and single, with 38% reporting no personal income. Also, 40% resided in Beirut, and 70% lived in urban settings. Around 32% were graduates, 30% were senior students, the majority were from private universities (76.8%) and the remainder were in their fourth year or earlier. Additionally, 55% were students, 44% did not work, and 10% had completed postgraduate education. The mean age of participants was 23.81 years (Table 1).

**Table 1.** Descriptive characteristics of the studied sample (N = 504)

Multinomial and Dichotomous Variables	Total	Students	Professional	*p*-value
	Frequency (%)	Frequency (%)	Frequency (%)	
Gender				
Male	146 (29.0%)	92 (27.1%)	54 (32.9%)	0.174
Female	358 (71.0%)	248 (72.9%)	110 (37.1%)	
Marital status				
Single	434 (86.1%)	323 (95.0%)	111 (67.7%)	<0.001
Married	66 (13.1%)	17 (5.0%)	53 (32.3%)	
Personal monthly income				
<250 USD	63 (12.5%)	238 (70.0%)	17 (10.4%)	<0.001
250–500 USD	112 (22.2%)	63 (18.5%)	49 (29.9%)	
>500 USD	38 (7.5%)	39 (11.5%)	98 (59.8%)	
Region of living				
Beirut	201 (39.9%)	129 (37.9%)	72 (43.9%)	0.367
Mount Lebanon	91 (18.1%)	68 (20.0%)	23 (14.0%)	
North	58 (11.5%)	36 (10.6%)	22 (13.4%)	
South	86 (17.1%)	59 (17.4%)	27 (16.5%)	
Beqaa	68 (13.5%)	48 (14.1%)	20 (12.2%)	
Living region category				
Rural	149 (29.6%)	240 (67.6%)	115 (32.4%)	0.914
Urban	355 (70.4%)	100 (67.1%)	49 (32.9%)	
Current university				
Public university	96 (19.0%)	47 (49.0%)	49 (51.0%)	<0.001
Private university	387 (76.8%)	291 (75.2%)	96 (24.8%)	
Foreign university	21 (4.2%)	2 (9.5%)	19 (90.5%)	
Current academic year				
First	11 (2.2%)			
Second	18 (3.6%)			
Third	36 (7.1%)			
Fourth	124 (24.6%)			
Fifth	135 (26.8%)			
Sixth	16 (3.2%)			
Graduate student	89 (17.7%)			
Not studying anymore	75 (14.9%)			
Highest level of education achieved				
I am an undergraduate student	277 (55.0%)			
Bachelor’s degree	117 (23.2%)			
PharmD	59 (11.7%)			
Master’s degree (MBA, MPH, etc.)	47 (9.3%)			
Doctorate (PhD, DBA, DPT, etc.)	3 (0.6%)			
Others	1 (0.2%)			
Employment status				
Full-time employee	128 (25.4%)	36 (28.1%)	92 (71.9%)	<0.001
Part-time employee	113 (22.4%)	75 (66.4%)	38 (33.6%)	
Self-employed	41 (8.1%)	19 (46.3%)	22 (53.7%)	
Do not work	222 (44.0%)	210 (94.6%)	12 (5.4%)	
Scale variables	Mean ± SD	Mean ± SD	Mean ± SD	
Age in years	23.81 ± 4.30	22.13 ± 2.35	27.30 ± 5.23	<0.001
Authentic Leadership Self-Assessment Questionnaire (ALSAQ-16) (Max /90)	57.26 ± 11.03	57.26 ± 10.51	57.26 ± 12.05	1.000
Global Self-Efficacy Questionnaire (GSE-10) (Max /50)	20.32 ± 5.73	20.14 ± 5.57	20.68 ± 6.04	0.323
Strategic Thinking Questionnaire (STQ-15) (Max/75)	51.63 ± 11.66	51.62 ± 11.71	51.63 ± 11.58	0.994
Public Service Motivation Scale (PSM-14) (Max/70)	50.68 ± 10.91	50.30 ± 11.09	51.46 ± 10.50	0.266
Pharmaceutical Policymaking Perception Scale (PPPS) (Max /100)	62.15 ± 8.38	62.45 ± 7.93	61.52 ± 9.21	0.268

A comparative analysis of pharmacy students and professionals revealed significant differences in several sociodemographic and socioeconomic characteristics, including age, marital status, income level and employment status (all *p* < 0.05). As expected, students were predominantly younger, single, with lower personal income, and more likely to be unemployed or working part-time, whereas professionals were older, more likely to be married, and predominantly employed full-time with higher income levels. No statistically significant variations were found between the two groups in their scores on the validated perception and motivation scales (all *p* > 0.05).

Participants scored above average on all concepts except for global self-efficacy, where the results were below average (Table 1). For the PPPS scale, the distribution showed that 142 participants (28.2%) scored less than 60, 190 (37.7%) scored between 60 and 65, 84 (16.7%) scored between 65 and 67, and 88 (17.5%) scored above 67. Notably, only 4% scored above 70.

Table 2 presents the construct validity and reliability measures for the scales used, including the newly developed PPPS. All scales demonstrated adequate construct validity (as evidenced by excellent KMO and Bartlett’s test results) and excellent reliability. The GSE-10 and PSM-14 items loaded on one factor, showing a unidimensional structure. The items of the new PPPS, ALSAQ-16, and STQ-15 loaded on two factors after Promax rotation. The two components of the PPPS reflect a positive (factor 1) and a negative (factor 2) current perception of pharmaceutical policymaking; the total represents 74% of the latent variable variance. The good structural validity of the PPPS is reflected in the correlation coefficient between its factors (r = 0.48; *p* < 0.001).

**Table 2.** Factor analyses using the Promax rotated matrix for bidimensional scales.

The Authentic Leadership Self-Assessment Questionnaire (ALSAQ-16)		
**Factor analysis with Promax rotation**	**Factor 1**	**Factor 2**
5. I can list my three greatest strengths	0.970	
1. I can list my three greatest weaknesses	0.863	
13. I accept the feelings I have about myself	0.807	
14. My morals guide what I do as a leader	0.799	
2. My actions reflect my core values	0.782	
6. I do not allow group pressure to control me	0.728	
7. I listen closely to the ideas of those who disagree with me	0.625	
15. I listen very carefully to the ideas of others before making decisions	0.594	
9. I seek feedback as a way of understanding who I really am as a person	0.575	
8. I let others know who I truly am as a person	0.539	
16. I admit my mistakes to others	0.464	
3. I seek others’ opinions before making up my own mind		0.868
11. I do not emphasize my own point of view at the expense of others		0.773
4. I openly share my feelings with others		0.703
12. I rarely present a ‘false’ front to others		0.703
10. Other people know where I stand on controversial issues		0.429
Percentage variance explained = 57.89%	50.63%	7.25%
Cronbach alpha = 0.933		
Kaiser-Meyer-Olkin (KMO) = 0.946		
Bartlett’s test of sphericity *p* < 0.001		
The General Self-Efficacy Scale (GSE-10)		
**Factor analysis**	**Factor 1**	
10. No matter what comes my way, I'm usually able to handle it	0.826	
6. I can solve most problems if I invest the necessary effort	0.825	
4. I am confident that I could deal efficiently with unexpected events	0.804	
5. Thanks to my resourcefulness, I know how to handle unforeseen situations	0.801	
8. When I am confronted with a problem, I can usually find several solutions	0.798	
9. If I am in a bind, I can usually think of something to do	0.792	
3. It is easy for me to stick to my aims and accomplish my goals	0.766	
1. I can always manage to solve difficult problems if I try hard enough	0.757	
7. I can remain calm when facing difficulties because I can rely on my coping abilities	0.712	
2. If someone opposes me, I can find means and ways to get what I want	0.688	
Percentage variance explained	60.53%	
Cronbach alpha = 0.927		
Kaiser-Meyer-Olkin (KMO) = 0.951		
Bartlett’s test of sphericity *p* < 0.001		
The Strategic Thinking Questionnaire (STQ-15)		
**Factor analysis with Promax rotation**	**Factor 1**	**Factor 2**
5. I try to understand how the facts in the situation are related to each other.	0.909	
4. I investigate the cause before taking action.	0.897	
2. I look for fundamental changes in the structure leading to significant improvements.	0.886	
3. I look at the ‘Big Picture’ in the information available before examining the details.	0.878	
1. I look for fundamental long-term corrective measures.	0.837	
15. I try to understand how a problem worked out after it was resolved.	0.827	
14. I stop and think about why I succeeded or failed	0.798	
12. I reconstruct an experience in my mind.	0.793	
11. I reconstruct an experience in my mind to understand how I feel about it.	0.769	
13. I consider how I could have handled the situation after it was resolved.	0.749	
9. I create a plan to solve a problem before considering other viewpoints.	0.471	
7. I ignore past decisions when considering current similar situations.		0.962
6. I ignore my past experiences when trying to understand situations presented to me.		0.903
8. I usually find only one explanation for the way things work.		0.879
10. I decide upon a point of view before I identify solutions to a problem.		0.637
Percentage variance explained = 70.44%	56.03%	14.41%
Cronbach alpha = 0.939		
Kaiser-Meyer-Olkin (KMO) = 0.931		
Bartlett’s test of sphericity *p* < 0.001		
Public Service Motivation Measurement (PSM-14)		
**Factor analysis with Promax rotation**	**Factor 1**	
5. Meaningful public service is very important to me.	0.875	
11. Serving other citizens would give me a good feeling even if no one paid me for it.	0.866	
4. I consider public service my civic duty.	0.856	
3. Seeing people get benefits from the public program deeply satisfies me.	0.844	
13. I am prepared to make enormous sacrifices for the good of society.	0.832	
8. I am often reminded by daily events how dependent we are on one another.	0.830	
9. I feel sympathetic to the plight of the underprivileged.	0.824	
1. I am interested in making public programs beneficial for my country/community	0.807	
10. To me, patriotism includes seeing to the welfare of others.	0.807	
12. Making a difference in society means more to me than personal achievements.	0.800	
2. Sharing my views on public policies with others is attractive to me.	0.798	
6. I would prefer the best for the whole community even if it harmed my interests.	0.771	
14. I believe in putting duty before self.	0.769	
7. It is difficult for me to contain my feelings when I see people in distress.	0.661	
Percentage variance explained	65.86%	
Cronbach alpha = 0.959		
Kaiser-Meyer-Olkin (KMO) = 0.960		
Bartlett’s test of sphericity *p* < 0.001		
Pharmaceutical Policymaking Perception Scale (PPPS)		

**Factor analysis with Promax rotation**	**Factor 1**	**Factor 2**
2. Policymakers in the pharmaceutical sector make decisions in a timely manner.	0.918	
3. Policymakers in the pharmaceutical sector foster research to gather the necessary information before making a decision.	0.902	
1. The national environment is adequate to make beneficial pharmaceutical policies.	0.879	
7. Policymakers in the pharmaceutical sector are true leaders.	0.853	
5. Policymakers in the pharmaceutical sector thoroughly evaluate the different consequences before making a final decision.	0.816	
6. Policymakers in the pharmaceutical sector are strategic thinkers.	0.761	
10. Policies currently implemented help the pharmaceutical sector reach its full potential.	0.757	
12. Pharmaceutical policies and laws should be updated every now and then according to the evolution of the field and the market.		0.920
13. Pharmacists should be more involved in policymaking.		0.918
9. There are policies available that are not implemented.		0.856
11. Changes in current policies can generate new opportunities.		0.852
8. The general population has lost faith that new policies will make any change.		0.797
Percentage variance explained = 73.87%	55.45%	18.41%
Cronbach alpha = 0.926		
Kaiser-Meyer-Olkin (KMO) = 0.930		
Bartlett’s test of sphericity *p* < 0.001		

Moreover, Table 3 displays the bivariate correlations among all measured concepts, supporting the concurrent and divergent validity of the PPPS. The PPPS showed positive but weak correlations with GSE and STQ, while its correlation with ALSAQ and PSM-14 was negative and non-significant. Significant differences were observed in scores across all PPPS quartiles (*p*-value for ANOVA < 0.001). The linear trend was significant for the association with STQ and GSE (*p* < 0.001 for both), but not with ALSAQ (p-linear trend = 0.09) or PSM-14 (p-linear trend = 0.345) (Supplemental Table S1).

**Table 3.** Spearman correlation coefficient between the scales used in the study

	ALSAQ-16	GSE-10	STQ-15	PSM-14	PPPS
	Correlation coefficient	Correlation coefficient	Correlation coefficient	Correlation coefficient	Correlation coefficient
ALSAQ-16	-				
GSE-10	0.465***	-			
STQ-15	0.489***	0.404***	-		
PSM-14	0.569***	0.434***	0.547***	-	
PPPS	−0.006	0.109*	0.117**	−0.039	-

* < 0.05; ** < 0.01; *** < 0.001 ALSAQ: Authentic Leadership Self-Assessment Questionnaire; GSE: The General Self-Efficacy Scale; STQ: Strategic Thinking Questionnaire; PSM-14: Public Service Motivation scale; PPPS: The Pharmaceutical Policymaking Perception Scale

Few personal and educational characteristics affected the measured concepts. Participants who did not work reported significantly lower GSE, while those had lower STQ scores. All other factors showed no significant effect on the measured concepts. Neither PSM-14 nor ALSAQ was associated with participants’ sociodemographic or educational characteristics (Supplemental Table S2).

Table 4 outlines the correlates of the PPPS. Type of pharmacist (student vs. working professional) did not show statistically significant associations with policymaking perceptions. Married participants had significantly higher PPPS scores. Additionally, higher GSE and STQ scores and lower PSM-14 scores were associated with higher PPPS. No significant associations were found with other variables, including age, gender, income, and ALSAQ (Table 4).

**Table 4.** Univariate ANOVA-GLM for the Pharmaceutical Policymaking Perception Scale (PPPS).

				95% Confidence Interval	
Parameters	Adjusted Mean EMM (SEM)	Adjusted Beta	*p*-value	Lower Bound	Upper Bound
Type of pharmacists					
Students	63.78 (0.76)	1.592	.133	−.489	3.674
Working pharmacists	62.19 (0.89)	Ref.			
Marital status					
Married	64.33 (1.14)	2.688	.029	.273	5.103
Single	61.64 (0.51)	Ref.			
Personal income					
Low income <250$	62.94 (0.86)	.517	.650	−1.723	2.758
Middle Income 250-500$	63.59 (0.93)	1.169	.294	−1.016	3.354
High income >500$	64.42 (0.89)	Ref.			
Gender					
Female	63.46 (0.63)	−.937	.257	−2.558	.684
Male	62.52 (0.87)	Ref.			
Region of dwelling					
Beirut	61.87 (1.34)	−.542	.640	−2.816	1.732
Mount Lebanon	61.43 (1.11)	−1.668	.213	−4.298	.962
North	64.77 (1.37)	2.670	.070	−.214	5.555
South	62.31 (1.20)	.019	.989	−2.605	2.643
Beqaa	62.30 (0.94)	Ref			
	Adjusted Correlation	Adjusted Beta	*P*-value	95% Confidence Interval	
Age in years		.020	.862	−.207	.247
ALSAQ-16	−0.065	−.067	.142	−.155	.022
GSE-10	0.031	.165	.040	.007	.322
STQ-15	0.032	.112	.007	.030	.193
PSM-14	−0.123	−.155	.001	−.246	−.064

* < 0.05; ** < 0.01; *** < 0.001 ALSAQ-16: Authentic Leadership Self-Assessment Questionnaire; GSE-10: The General Self-Efficacy Scale; STQ-15: Strategic Thinking Questionnaire; PSM-14: Public Service Motivation scale.

## Discussion

This study aimed to develop and validate a tool to assess early-career pharmacists’ perceptions of pharmaceutical policymaking in Lebanon. The newly developed PPPS demonstrated excellent psychometric properties, with its items loaded on two factors representing the positive and negative perceptions of pharmaceutical policymaking. This bifactorial structure offers valuable insights into pharmacists’ views, enabling a more comprehensive assessment of policy perceptions. The scale demonstrated excellent reliability as well as robust content, construct, structural, and concurrent validity, indicating consistency in measurement (Boateng et al., 2018).

To the best of current knowledge, no tool exists in the literature to measure this critical concept. The results suggest that the PPPS scale can effectively assess pharmacists’ perceptions of policymaking in Lebanon, providing policymakers with a means to evaluate their performance and the effectiveness of policies in a country facing multiple crises and complex needs. Additionally, the scale could identify areas requiring improvement and track changes over time. While the internal validity of the scale is robust, expanding the sample to include other pharmacists and measuring its test-retest reliability are recommended to confirm its external validity and reliability and ability to detect changes or maintain stability over time. Furthermore, the scale should be cross-culturally adapted for use in other countries and among other stakeholders within the healthcare system (Lilly et al., 2023).

Since the selected sample adequately represented Lebanon’s pharmaceutical workforce demographics (Hallit et al., 2017), with female predominance and proportional representation across geographical regions and educational institutions (Alameddine et al., 2019), the generalizability of findings to early-career pharmacists’ perceptions of the measured constructs is enhanced. Overall, the distribution of the PPPS was as follows: 28.2% had less than 60, while only 4% scored above 70, showing a relatively low perception of pharmaceutical policymaking in Lebanon. This low perception could stem from policymaking inadequacy in the current context, lack of trust, skepticism, or lack of communication between authorities and early-career pharmacists (Transparency International, 2016).

This study also explored early-career pharmacists’ perceptions of leadership, self-efficacy, strategic thinking, and public service motivation, using several validated tools within the Lebanese context and linking these concepts to pharmaceutical policymaking. All the validated scales demonstrated robust psychometric properties, with high validity and reliability across all measures. While early-career pharmacists rated themselves slightly above average in leadership and strategic thinking, their general self-efficacy was notably below average. This finding indicates a critical gap in self-confidence that could hinder their effectiveness as future leaders in the field, highlighting the importance of developing such skills among early-career pharmacists in Lebanon at the undergraduate level by incorporating related courses into pharmacy curricula, continuing education programs, and professional development activities (Hajj et al., 2024; Popovich et al., 2007; Sacre et al., 2024). This approach would help pharmacists assume their evolving role and drive healthcare transformation toward better patient health (Ali et al., 2022).

There were no statistically significant differences in the validated perception and motivation scale scores between professionals and student pharmacists. Both groups seemed to have comparable perceptions of pharmaceutical policies, leadership, self-efficacy, and public service motivation, despite variations in age, income, and work status. This finding may reflect the professional principles and common academic preparation that are established in pharmacy practice and education. It also indicates that preparedness to participate in policymaking may emerge early and remain consistent throughout various stages of professional growth, rather than being solely influenced by career stage (Hajj et al., 2023).

Notably, minimal demographic or educational factors influenced the measured concepts, with non-working pharmacists exhibiting the lowest self-efficacy. The lower GSE scores among non-working pharmacists suggest that practical experience plays a crucial role in developing professional confidence, supporting previous research highlighting the importance of experiential learning in pharmacy education and suggesting the need for some institutions to enhance the competencies taught in these domains. GSE has been associated with paid practicum, extended experiential learning, openness to change, and higher grade point averages (GPAs) among pharmacy students (Jacobs et al., 2019; Yorra, 2014). Among health practitioners, it has been correlated with resilience, adaptability, job satisfaction, well-being, leadership, and stress management skills (Johnson et al., 2020; Magon et al., 2023; Milam et al., 2019; Yao et al., 2014).

In contrast, both public service motivation (PSM-14) and leadership (ALSAQ-16) appeared unaffected by any sociodemographic or educational characteristics, underscoring the stability of these traits across various backgrounds among the study population. The absence of association with the academic year, the achieved educational degree, or the university further confirms the deep need to tackle these competencies in all educational institutions (Zeenny et al., 2021); encouraging participation in professional organizations, emphasizing the social role of pharmacy, and providing opportunities for community engagement are to be promoted by educators (Fernandes et al., 2022).

Married participants, first-year students, students from the North, and BAU students had significantly higher PPPS scores, whereas LIU students scored lower. The higher scores among first-year students may reflect more optimistic views toward pharmaceutical policymaking than their peers. This trend may stem from recent advancements in teaching methodologies implemented in schools to meet modern educational demands, which emphasize self-directed learning and critical thinking. Such approaches offer first-year students an engaging curriculum that encourages independent exploration of pharmaceutical policies, thereby fostering their sense of agency and optimism regarding their potential roles in healthcare decision-making (Vianna et al., 2024). However, the observed differences in the understanding of the Lebanese policymaking context underscore the importance of addressing gaps and enhancing policymaking perceptions across all categories of pharmacists.

Moreover, higher self-efficacy and strategic thinking were associated with a higher PPPS, which could be linked to subjective positivity in self-rating or an actual willingness to engage in policymaking among confident pharmacists. The first possibility could be linked to a confirmation bias, where individuals assessing themselves become more self-aware and selectively recall instances that confirm their self-image (Peters, 2022). Nevertheless, although a higher self-efficacy has been associated with the willingness of pharmacists to engage in new tasks in general (Martin et al., 2010), a lower public service motivation was found to be associated with higher PPPS scores.

The positive association between self-efficacy and perceptions of pharmaceutical policymaking engagement aligns with prior studies (Ehidiamen Oamen, 2022; Kalungia et al., 2021; Noureldin et al., 2021). Similarly, strategic thinking was found to be significantly associated with perceptions of engagement in pharmaceutical policymaking. This finding is supported by previous research in pharmacy and health professions education, which underscores strategic thinking as essential for health leadership and innovation, in addition to clinical competencies (Castleberry et al., 2023; Fang et al., 2011; Noureldin & Melton, 2024; Salavati et al., 2017; Zeineddine et al., 2023). These abilities and policy engagement are also related, which aligns with competency-based education frameworks that emphasize the integration of personal leadership and management skills into pharmacy training, including the FIP Global Competency Framework and its Lebanese adaptation by the OPL (Hajj et al., 2022).

No significant associations were observed with other variables, such as age, gender, income, and leadership. The less favorable views about policymaking expressed by pharmacists with higher public service motivation, regardless of their leadership skills, reflect an alarming disconnection between motivation and policy engagement, potentially leading to reduced policy input from highly motivated pharmacists. This situation may also lead to policies that do not fully leverage pharmacists’ public health potential, underutilize pharmacists’ expertise, and create additional frustration among service-oriented pharmacists who might feel disconnected from policy decisions (Agomo et al., 2020). This disconnect may indicate cognitive dissonance, where actions contradict beliefs, leading to feelings of guilt or shame and, ultimately, further disengagement. Empowering pharmacists with the necessary skills and enhancing communication within the pharmaceutical sector are required to alleviate the situation, given the particularly challenging Lebanese context characterized by multifaceted crises.

### Public health implications

These results highlight a critical gap between pharmacists’ public service motivation and their engagement with policymaking. Addressing this disconnection by both educators and policymakers could significantly enhance pharmacists’ contributions to public health and lead to more effective health policies that fully leverage pharmacists’ unique position in the healthcare system. There is an urgent need to integrate leadership, self-efficacy, and strategic thinking abilities into educational curricula, especially during economic hardships, as strategic thinking equips pharmacy professionals with the mindset necessary to make sound decisions and effectively utilize available resources.

These findings should prompt pharmacy organizations to create more opportunities for policy engagement among motivated pharmacists, develop leadership programs that emphasize policy involvement, and establish clearer pathways for pharmacists to contribute to policymaking. By fostering leadership and strategic thinking in pharmacy education, future pharmacists can substantially contribute to policymaking, suggest new regulations, advocate for changes in the profession, and adapt to Lebanon’s dynamic healthcare environment. An inclusive strategy will guarantee the continued growth and advancement of the pharmacy profession in Lebanon, enabling pharmacy students and young pharmacists to become capable leaders and visionary strategic thinkers.

### Strengths and limitations

One of the study’s strengths is the diverse sample obtained through social media, which improves the generalizability of the findings. The use of validated measures increases the credibility of the results and reduces the risk of information bias. Moreover, the focus on recent data reflects current trends and perceptions among early-career pharmacists in Lebanon. Additionally, applying multivariable analysis methods helps to reduce the potential for confounding bias.

However, the study has also several limitations. Its cross-sectional design limits the ability to conclude causality, and reliance on self-reported data may introduce biases, particularly recall bias, confirmation bias, or social desirability bias. Selection bias may have occurred, as participation was voluntary and may not fully represent the broader population of pharmacy students and professionals in Lebanon. While combining pharmacy students and working professionals allowed for an examination of general trends across the field, this heterogeneity introduces variability related to differences in experience, exposure, and professional status, which may have influenced the findings despite subgroup analyses. The online data collection method, combined with a lengthy questionnaire, may have led to participant fatigue, potentially affecting the accuracy of responses. Although this type of bias is expected to be non-differential, it may still underestimate certain non-significant relationships. Furthermore, the sample may not fully represent the population, as it excludes pharmacists without internet access. Preliminary validation was conducted using exploratory factor analysis and internal consistency measures, full formal validation including confirmatory factor analysis and test-retest reliability was not performed. Therefore, future studies using face-to-face interviews with a more diverse sample, including both early-career and more experienced pharmacists, are recommended to validate these findings and address the specific challenges of the Lebanese context. Furthermore, a broader consideration of educational, cultural, social, and economic factors is recommended to include more pharmacists and optimize the profession.

## Conclusion

This study developed and validated the Pharmaceutical Policymaking Perception Scale, which demonstrated excellent psychometric properties. Policymaking perceptions varied among early-career pharmacists, showing positive correlations with self-reported general self-efficacy and strategic thinking. However, pharmacists with public service motivation had lower perceptions, regardless of their leadership skills. The potential disconnect between the studied concepts is concerning. We recommend that pharmacy education programs in Lebanon integrate targeted training modules focused on health policy, leadership, and strategic thinking to better prepare students for active engagement in pharmaceutical policymaking. Improved collaboration between academia and regulatory institutions is essential to better prepare pharmacists for active roles in health policy development in Lebanon. Further research is recommended to confirm these findings, and urgent action by educators and policymakers is essential to effectively engage with early-career pharmacists and enhance their motivation to serve the profession in challenging circumstances.

## Supplementary Material

Supplementary Tables_Clean.docx
