# Supplementary material for: Young pharmacists as tomorrow’s decision-makers: tool validation and perceptions of pharmaceutical policymaking in Lebanon
Source: J Pharm Policy Pract. 2025 Dec 23;19(1):2600242. doi: 10.1080/20523211.2025.2600242 (PMC12777755; doi:10.1080/20523211.2025.2600242)
Supplement: Supplementary Tables_Clean.docx [file JPPP_A_2600242_SM3201.docx]

**Supplementary Tables**

**Table S1.** Scales distribution according to PPPS quartiles

| **Pharmaceutical Policymaking Scale (PPPS) Quartiles** | | **N** | **Mean** | **Std. Deviation** | **Std. Error** | **95% Confidence Interval**  **for Mean** | | **Minimum** | **Maximum** |
| --- | --- | --- | --- | --- | --- | --- | --- | --- | --- |
|  |  |  |  |  |  | **Lower Bound** | **Upper Bound** |  |  |
| Authentic Leadership Self-Assessment Questionnaire over 90 | ≤60.00 | 142 | 59.3873 | 8.60138 | 0.72181 | 57.9604 | 60.8143 | 21.00 | 80.00 |
|  | 60.01 - 65.00 | 190 | 53.5526 | 11.74621 | 0.85216 | 51.8717 | 55.2336 | 16.00 | 80.00 |
|  | 65.01 - 66.67 | 84 | 59.0476 | 11.63077 | 1.26902 | 56.5236 | 61.5717 | 21.00 | 80.00 |
|  | 66.68+ | 88 | 60.1364 | 10.27460 | 1.09528 | 57.9594 | 62.3133 | 26.00 | 80.00 |
|  | Total | 504 | 57.2619 | 11.02784 | 0.49122 | 56.2968 | 58.2270 | 16.00 | 80.00 |
| General Self-efficacy over 50 | ≤60.00 | 142 | 20.6690 | 5.06374 | 0.42494 | 19.8289 | 21.5091 | 5.00 | 30.00 |
|  | 60.01 - 65.00 | 190 | 18.5632 | 6.22962 | 0.45194 | 17.6717 | 19.4547 | 0.00 | 30.00 |
|  | 65.01 - 66.67 | 84 | 21.6310 | 5.09377 | 0.55578 | 20.5255 | 22.7364 | 6.00 | 30.00 |
|  | 66.68+ | 88 | 22.2955 | 5.16656 | 0.55076 | 21.2008 | 23.3901 | 9.00 | 30.00 |
|  | Total | 504 | 20.3194 | 5.73045 | 0.25525 | 19.8179 | 20.8209 | 0.00 | 30.00 |
| Strategic Thinking Questionnaire over 75 | ≤60.00 | 142 | 51.8099 | 9.04593 | 0.75912 | 50.3091 | 53.3106 | 27.00 | 75.00 |
|  | 60.01 - 65.00 | 190 | 48.1105 | 11.45561 | 0.83108 | 46.4711 | 49.7499 | 15.00 | 75.00 |
|  | 65.01 - 66.67 | 84 | 55.1905 | 13.31950 | 1.45328 | 52.3000 | 58.0810 | 15.00 | 75.00 |
|  | 66.68+ | 88 | 55.5341 | 12.02292 | 1.28165 | 52.9867 | 58.0815 | 17.00 | 75.00 |
|  | Total | 504 | 51.6290 | 11.66170 | 0.51945 | 50.6084 | 52.6495 | 15.00 | 75.00 |
| Public Service Motivation Scale over 70 | ≤60.00 | 142 | 53.7394 | 9.45664 | 0.79358 | 52.1706 | 55.3083 | 21.00 | 70.00 |
|  | 60.01 - 65.00 | 190 | 46.5737 | 11.22225 | 0.81415 | 44.9677 | 48.1797 | 14.00 | 70.00 |
|  | 65.01 - 66.67 | 84 | 52.0595 | 10.22441 | 1.11557 | 49.8407 | 54.2784 | 21.00 | 70.00 |
|  | 66.68+ | 88 | 53.3182 | 10.44586 | 1.11353 | 51.1049 | 55.5314 | 14.00 | 70.00 |
|  | Total | 504 | 50.6845 | 10.91143 | 0.48603 | 49.7296 | 51.6394 | 14.00 | 70.00 |

**Table S2.** Multivariable analysis of measured concepts using the GLM method

|  | **Beta** | **P-value** | | **Confidence interval** | |
| --- | --- | --- | --- | --- | --- |
|  |  |  |  | **Lower** | **Upper** |
| **Dependent variable: Authentic Leadership Self-Assessment Questionnaire** | | | | | |
| Gender (female vs. male*) | 1.303 | | 0.253 | -0.935 | 3.542 |
| Marital status (Married vs. single*) | -1.754 | | 0.298 | -5.062 | 1.553 |
| Highest education (undergraduate) | 1.365 | | 0.426 | -2.005 | 4.735 |
| Highest education (BS) | 2.503 | | 0.112 | -0.586 | 5.592 |
| Income (low) | 1.360 | | 0.428 | -2.012 | 4.731 |
| Income (middle) | 2.358 | | 0.119 | -.608 | 5.324 |
| Employment status (Full-time vs. self-employed*) | 1.237 | | 0.561 | -2.943 | 5.417 |
| Employment status (I do not work vs. self-employed*) | -0.478 | | 0.831 | -4.869 | 3.913 |
| Employment status (Part-time vs. self-employed*) | 1.616 | | 0.455 | -2.630 | 5.861 |
| Region (Beirut vs. South*) | -0.243 | | 0.871 | -3.168 | 2.683 |
| Region (Beqaa vs. South*) | -2.295 | | 0.212 | -5.908 | 1.317 |
| Region (Mount Lebanon vs. South*) | -0.589 | | 0.734 | -3.986 | 2.809 |
| Region (North vs. South*) | -0.712 | | 0.709 | -4.456 | 3.031 |
| Living place (rural vs. urban*) | -0.285 | | 0.810 | -2.607 | 2.038 |
| Age in years | 0.115 | | 0.504 | -0.223 | 0.453 |
| **Dependent variable: The General Self-Efficacy Scale** | | | | | |
| Gender (female vs. male*) | -0.063 | | 0.915 | -1.219 | 1.094 |
| Marital status (Married vs. single*) | -0.746 | | 0.392 | -2.455 | 0.963 |
| Highest education (undergraduate) | -0.682 | | 0.442 | -2.423 | 1.060 |
| Highest education (BS) | -0.255 | | 0.754 | -1.851 | 1.342 |
| Income (low) | -1.398 | | 0.115 | -3.141 | 0.344 |
| Income (middle) | -0.073 | | 0.925 | -1.606 | 1.460 |
| Employment status (Full-time vs. self-employed*) | -2.108 | | 0.056 | -4.269 | 0.052 |
| **Employment status (I do not work vs. self-employed*)** | **-2.288** | | **0.048** | **-4.557** | **-0.019** |
| Employment status (Part-time vs. self-employed*) | -1.487 | | 0.183 | -3.681 | 0.707 |
| Region (Beirut vs. south*) | -0.820 | | 0.287 | -2.332 | 0.692 |
| Region (Beqaa vs. South*) | -1.660 | | 0.081 | -3.527 | 0.207 |
| Region (Mount Lebanon vs. South*) | -1.630 | | 0.069 | -3.386 | 0.126 |
| Region (North vs. South*) | -1.145 | | 0.245 | -3.080 | 0.789 |
| Living place (rural vs. urban*) | 0.353 | | 0.563 | -0.847 | 1.554 |
| Age in years | -0.135 | | 0.130 | -0.310 | .040 |
| **Dependent variable: Strategic Thinking Questionnaire** | | | | | |
| Gender (female vs. male*) | 0.281 | | 0.816 | -2.089 | 2.652 |
| Marital status (Married vs. single*) | -1.910 | | 0.284 | -5.412 | 1.592 |
| Highest education (undergraduate) | 0.131 | | 0.943 | -3.438 | 3.700 |
| Highest education (BS) | 1.801 | | 0.280 | -1.470 | 5.073 |
| Income (low) | -1.474 | | 0.418 | -5.044 | 2.097 |
| Income (middle) | 0.115 | | 0.943 | -3.026 | 3.255 |
| Employment status (Full time vs. self-employed*) | -0.734 | | 0.745 | -5.161 | 3.692 |
| Employment status (I do not work vs. self-employed*) | -1.420 | | 0.549 | -6.070 | 3.229 |
| Employment status (Part time vs. self-employed*) | -1.115 | | 0.626 | -5.611 | 3.380 |
| Region (Beirut vs. south*) | -0.730 | | 0.644 | -3.828 | 2.368 |
| Region (Beqaa vs. south*) | -0.365 | | 0.851 | -4.190 | 3.460 |
| Region (Mount Lebanon vs. south*) | -1.090 | | 0.552 | -4.688 | 2.508 |
| Region (North vs. south*) | -1.510 | | 0.455 | -5.474 | 2.455 |
| Living place (rural vs. urban*) | 1.166 | | 0.352 | -1.293 | 3.626 |
| Age in years | -0.247 | | 0.176 | -0.605 | 0.111 |
| **Dependent variable: Public Service Motivation** | | | | | |
| Gender (female vs. male*) | -0.805 | | 0.470 | -2.993 | 1.383 |
| Marital status (Married vs. single*) | 0.101 | | 0.951 | -3.132 | 3.334 |
| Highest education (undergraduate) | -0.440 | | 0.793 | -3.734 | 2.854 |
| Highest education (BS) | 1.425 | | 0.354 | -1.594 | 4.444 |
| Income (low) | -0.709 | | 0.673 | -4.005 | 2.586 |
| Income (middle) | 0.526 | | 0.722 | -2.373 | 3.425 |
| Employment status (Full-time vs. self-employed*) | -2.402 | | 0.249 | -6.488 | 1.684 |
| Employment status (I do not work vs. self-employed*) | -3.427 | | 0.117 | -7.719 | 0.865 |
| Employment status (Part-time vs. self-employed*) | -2.877 | | 0.174 | -7.027 | 1.273 |
| Region (Beirut vs. South*) | 0.299 | | 0.837 | -2.560 | 3.159 |
| Region (Beqaa vs. South*) | -0.777 | | 0.666 | -4.308 | 2.754 |
| Region (Mount Lebanon vs. South*) | 2.699 | | 0.111 | -0.622 | 6.021 |
| Region (North vs. South*) | 0.455 | | 0.807 | -3.204 | 4.115 |
| Living place (rural vs. urban*) | 0.779 | | 0.500 | -1.491 | 3.050 |
| Age in years | -0.280 | | 0.097 | -0.610 | 0.051 |
| **Dependent Variable: Pharmaceutical Policymaking Perception Scale** | | | | | |
| Gender (female vs. male*) | 1.463 | | 0.085 | -0.204 | 3.130 |
| Marital status (Married vs. single*) | 2.372 | | 0.059 | -0.091 | 4.836 |
| Highest education (undergraduate) | 1.966 | | 0.125 | -0.544 | 4.476 |
| Highest education (BS) | 1.315 | | 0.262 | -0.986 | 3.615 |
| Income (low) | 0.165 | | 0.897 | -2.346 | 2.676 |
| Income (middle) | 0.897 | | 0.426 | -1.313 | 3.106 |
| Employment status (Full-time vs. self-employed*) | 1.101 | | 0.488 | -2.013 | 4.214 |
| Employment status (I do not work vs. self-employed*) | 1.054 | | 0.527 | -2.217 | 4.325 |
| Employment status (Part-time vs. self-employed*) | 0.524 | | 0.745 | -2.638 | 3.686 |
| Region (Beirut vs. south*) | 0.036 | | 0.974 | -2.143 | 2.215 |
| Region (Beqaa vs. South*) | -0.294 | | 0.830 | -2.985 | 2.396 |
| Region (Mount Lebanon vs. south*) | -1.313 | | 0.309 | -3.844 | 1.218 |
| Region (North vs. South*) | 1.952 | | 0.170 | -0.836 | 4.741 |
| Living place (rural vs. urban*) | 0.555 | | 0.529 | -1.175 | 2.285 |
| Age in years | 0.035 | | 0.786 | -0.217 | 0.287 |
| *Reference group | | | | | |
